# The Effectiveness of Telerehabilitation for Functional Recovery After Orthopedic Surgery: A Systematic Review and Meta-Analysis

**DOI:** 10.1089/tmr.2023.0057

**Published:** 2024-03-27

**Authors:** Mattia Morri, Riccardo Ruisi, Antonio Culcasi, Vincenzo Peccerillo

**Affiliations:** IRCCS Istituto Ortopedico Rizzoli, Servizio di Assistenza Infermieristica, tecnica e della riabilitazione, Bologna, Italia.

**Keywords:** telerehabilitation, meta-analysis, systematic literature review, orthopedic surgery

## Abstract

**Objective:**

The aim of this systematic review is to evaluate the effectiveness of physiotherapy treatment provided remotely via the Internet in the postoperative treatment of orthopedic patients and compare it with standard physiotherapy (face-to-face treatment or home-based treatment) in terms of motor performance, pain symptoms, and functional recovery.

**Methods:**

A systematic search of MEDLINE, Physiotherapy Evidence Database; EMBASE, SCOPUS, and CINHAL was conducted. Two independent reviewers performed study selection, data extraction, risk of bias (ROB) assessment using Cochrane ROB 2 tools, and summarize the results by Grading of Recommendations Assessment, Development, and Evaluation.

**Results:**

Eleven randomized controlled trial were selected. Pooled results showed improvement in motor performance in favor of the telerehabilitation group at 4–6 weeks (standardized mean difference −0.24, 95% confidence interval −0.45, −0.02, *p* = 0.03), and these differences were close to the minimum clinically important difference for Time Up and Go test. For pain and functional recovery, the results showed differences not statistically important. The certainty of evidence ranged from moderate to very low.

**Conclusion:**

For joint replacement patients, treatment conducted via telerehabilitation appears able to provide levels of motor performance better to that achieved through home-based treatment. In contrast, conclusive evidence that telerehabilitation is comparable to standard face-to-face treatment are not available.

## Introduction

In recent years, the focus on telerehabilitation has grown significantly. The U.S. National Library of Medicine introduced the medical subject heading (MeSH) term “telerehabilitation” in 2016, defining it as the delivery of therapeutic rehabilitation treatment at a distance using telecommunication technologies.^1^ From 2016 to date, the number of publications referring to this term totaled 673, rising from 71 in 2016 to 168 in 2020 and 211 in 2021. Telerehabilitation was introduced to manage patients suffering from various diseases, such as chronic respiratory, cardiovascular, and neurological diseases.^2–4^ On the basis of reviews of the available scientific literature, several authors^5–8^ concluded that, for the field of rehabilitation, this approach was to be considered promising but that there was insufficient evidence regarding its effectiveness. Seron^6^ emphasized the need for better quality systematic reviews as well.

In examining the possible role of telerehabilitation in the treatment of musculoskeletal dysfunctions, Turolla et al.^9^ emphasized the potential of this approach and the need for practitioners to thoroughly investigate the most appropriate usage modalities, envisaging the use of telerehabilitation as the sole method of treatment or as integrated with face-to-face treatment. In the context of orthopedic surgery patients, in 2017, Pastora Bernal et al.^10^ underscored the strong evidence in favor of telerehabilitation for treating patients who had undergone hip and knee implantation and, at the same time, the need for further studies targeting different types of orthopedic surgery.

Therefore, the aim of this systematic review is to evaluate the effectiveness of physiotherapy treatment provided remotely via the Internet in the postoperative treatment of orthopedic patients and compare it with standard physiotherapy (face-to-face treatment or home-based treatment) in terms of motor performance, pain symptoms, and functional recovery.

## Materials and Methods

This systematic review was drafted according to the guidelines of the PRISMA Checklist.^11^ Before being performed, the protocol was registered in the PROSPERO international database (registration number CRD42021267922). A search of the literature was conducted by consulting MEDLINE, Physiotherapy Evidence Database, Scopus, CINHAL, and Embase with no limitation of language or year of publication. In addition, the bibliographies of the articles found during the search were also taken into account and a manual search was conducted using Google Scholar. The search string was constructed by combining MeSH terms and free search terms. The search strings were adapted to the characteristics of the different search engines consulted. Table 1 summarizes the search strings used.

**Table 1.** Search Strategy for Each Database and Results

Database	Search strategy	Results
Medline	(((((((((((((“Telemedicine”[Mesh]) OR (“Internet-Based Intervention”[Mesh])) OR (telerehabilitation[Title/Abstract])) OR (digital health[Title/Abstract])) OR (ehealth[Title/Abstract])) OR (“mobile health”[Title/Abstract])) OR (“mhealth”[Title/Abstract])) OR (“telecare”[Title/Abstract])) OR (internet based rehabilitation[Title/Abstract])) OR (internet based intervention[Title/Abstract])) OR (((Web-Based Exercise[Title/Abstract]) OR (therapy computer assisted[Title/Abstract])) OR (“videoconferencing”[Title/Abstract]))) OR (“mobile application”[Title/Abstract])) OR (Virtual Rehabilitations[Title/Abstract])) AND ((((“orthopedic surgery”[Title/Abstract])) OR (“musculoskeletal diseases”[Title/Abstract])) OR ((“Orthopedic Procedures”[Mesh]) OR “Musculoskeletal Diseases”[Mesh])) Filters: Clinical Study, Clinical Trial, Observational Study, Randomized Controlled Trial	280
Embase	telemedicine:ti,ab,kw OR “web-based intervention”:ti,ab,kw OR telerehabilitation:ti,ab,kw OR “digital health”:ti,ab,kw OR telehealth:ti,ab,kw OR “mobile health application”:ti,ab,kw OR mhealth:ti,ab,kw OR telecare:ti,ab,kw OR “computer assisted therapy”:ti,ab,kw OR videoconferencing:ti,ab,kw OR “mobile application”:ti,ab,kw OR “virtual rehabilitations”:ti,ab,kw AND “orthopedic surgery”:ti,ab,kw OR “musculoskeletal diseases”:ti,ab,kw OR arthroplasty:ti,ab,kw OR “spine surgery”:ti,ab,kw	396
Central	(“telemedicine”):ti,ab,kw OR (“telerehabilitation”):ti,ab,kw OR (internet based intervention):ti,ab,kw OR (digital health):ti,ab,kw OR (mobile application):ti,ab,kw” AND (orthopedic surgery):ti,ab,kw OR (musculoskeletal diseases):ti,ab,kw	277
Scopus	(((TITLE-ABS-KEY (telemedicine) OR TITLE-ABS-KEY (telerehabilitation) OR TITLE-ABS-KEY (internet AND based AND rehabilitation) OR TITLE-ABS-KEY (web-based AND exercise) OR TITLE-ABS-KEY (videoconferencing) OR TITLE-ABS-KEY (mobile AND application) OR TITLE-ABS-KEY (digital AND health) OR TITLE-ABS-KEY (mhealth) OR TITLE-ABS-KEY (ehealth) OR TITLE-ABS-KEY (mobile AND health) OR TITLE-ABS-KEY (internet AND based AND rehabilitation) OR TITLE-ABS-KEY (virtual AND rehabilitations))) AND ((TITLE-ABS-KEY (orthopedic AND surgery) OR TITLE-ABS-KEY (musculoskeletal AND diseases)))) AND ((TITLE-ABS-KEY (clinical AND trial) OR TITLE-ABS-KEY (observational AND study)))	275
CINHAL	telemedicine OR telerehabilitation OR internet based intervention OR mobile application OR internet based rehabilitation OR Web-Based Exercise OR digital health OR videoconferencing AND orthpedic surgery OR musculoskeletal diseases	117
PEDro	Telerehabilitation; clinical trial	110

PEDro, Physiotherapy Evidence Database.

The search was conducted from inception until November 11, 2023. Two reviewer independently screened titles, abstracts, and text of the manuscripts for potentially eligible studies. When the two reviewers could not reach an agreement, a third auditor was consulted to make a decision. The selection of articles was conducted using Rayyan Intelligent Systematic Review application (https://www.rayyan.ai/).^12^

### Inclusion and exclusion criteria

All randomized controlled trials were included to evaluate the benefits obtained with the physiotherapy treatment administered. Functional abilities and pain could be presented as both primary and secondary outcomes of the study.

Participants in the study could be adolescents (under 18 years of age), adults (over 18 years of age), and senior citizens (over 65 years of age) who had undergone a surgical procedure for an orthopedic condition. All types of surgery were considered. Patients diagnosed with musculoskeletal disorders who were treated conservatively without surgical treatment were excluded. Studies that compared intervention with standard physiotherapy were included.

Intervention treatment was defined as physiotherapy administered remotely through the use of web technology with the aid of a smartphone or other similar tools. Examples of interventions were videoconferences, applications that could enable the patient to independently perform exercises and treatment plans, and rehabilitation interventions performed remotely with the use of virtual reality. Studies involving a single remote visit or telephone contact were not included. Standard physiotherapy was understood as the face-to-face administration of physiotherapy treatment with physical contact between physical therapist and patient or as an exercise program performed by patient at home without supervision. Evaluation of the outcomes achieved was to take place between 4 and 6 weeks after surgery.

### Outcome

Motor performance, pain, and functional recovery examined, with a follow-up at 4–6 weeks after surgery, were considered as outcomes.

Outcomes related to motor performance were measured with a specific objective test such as Time Up and Go (TUG) and Walking Speed. Pain symptoms were measured with visual-analogue scale or numeric rating scale. Functional recovery was measured with objective, patient-oriented assessment scales such as Western Ontario and McMaster Universities Osteoarthritis Index, Knee Injury and Osteoarthritis Outcome Score, Hip Knee Disability and Osteoarthritis Outcome Score, and International Knee Documentation Committee.

### Data extraction

A structured checklist, planned before the beginning of the study, was used to retrieve data from the studies included by two independently reviewers. The data collected from each article related to the setting, patient age, surgical procedure applied, size of the sample studied, details of the intervention treatment, outcomes taken into account, and timing thereof. The extraction of quantitative data for the purposes of the meta-analysis involved the collection of the following data for the intervention group and the control group: posttreatment means, pre–posttreatment mean difference (MD), MD control group—group of intervention, standard deviation, standard error, *p*-value, and *t*-test value. In the event that it would not be possible to find all the necessary data from the published article, to proceed with the meta-analysis, an e-mail was sent to the corresponding author of the article. Where possible, not available data were calculated by the software application of RavMan 5.4 (RevMan; The Cochrane Collaboration, London, UK)

### Risk of bias analysis

Two reviewers independently performed the risk of bias (ROB) analysis for each study included in the review. The *Cochrane Handbook for Systematic Reviews of Interventions* was taken as reference for the analysis.^13^ The following domains were considered: randomization process, deviations from the intended intervention, handling of missing data, outcome measurement methods, and data return methods. When discrepancies in evaluation emerged, these were discussed by the two reviewers and, if necessary, a third reviewer was involved. ROB graph was created through RobVis visualization tool.^14^

### Data synthesis strategy

A descriptive summary of the data extracted from the studies was provided. Data from studies involving the same outcomes considered with the same measurement times were summed together via a meta-analysis which, given the continuous nature of the outcomes considered, used the MD or standardized mean difference where necessary. Data were analyzed with Review Manager 5.4 software. Based on the clinical heterogeneity of the intervention and the conventional treatment that could be hypothesized, a random effect approach was used to analyze the data. Pooled estimation for the different outcome and for different subgroups was conducted if it was possible to include at least three studies.

Data heterogeneity was analyzed using the I2 test. Heterogeneity was considered substantial when the I2 exceeded 50%. To explain the possible study heterogeneity, a subgroup analysis was planned taking into account the age of the study population (adolescents, adults, and the elderly), type of surgery (hip, knee, shoulder, spinal surgery, or other), the type of outcome used, and the type of comparator (face-to-face treatment and home-based physiotherapy). Sensitivity analysis based on methodological quality was conducted by removing studies presenting a ROB related to handling randomization and missing data. The decision was made to analyze publication bias using the Funnel Plot if the meta-analysis included at least 10 studies.^15^

The Grading of Recommendations Assessment, Development, and Evaluation (GRADE) method was applied to evaluate and summarize the evidence, while the GradePRO programme was used to construct the “Summary of findings” table.^16^ The table shows the data for the three outcomes taken into account in the review, reporting the overall quality of the evidence from the selected studies and the magnitude of the effect produced by the intervention examined.

## Results

### Data selection and retrieval process

**FIG. 1.**
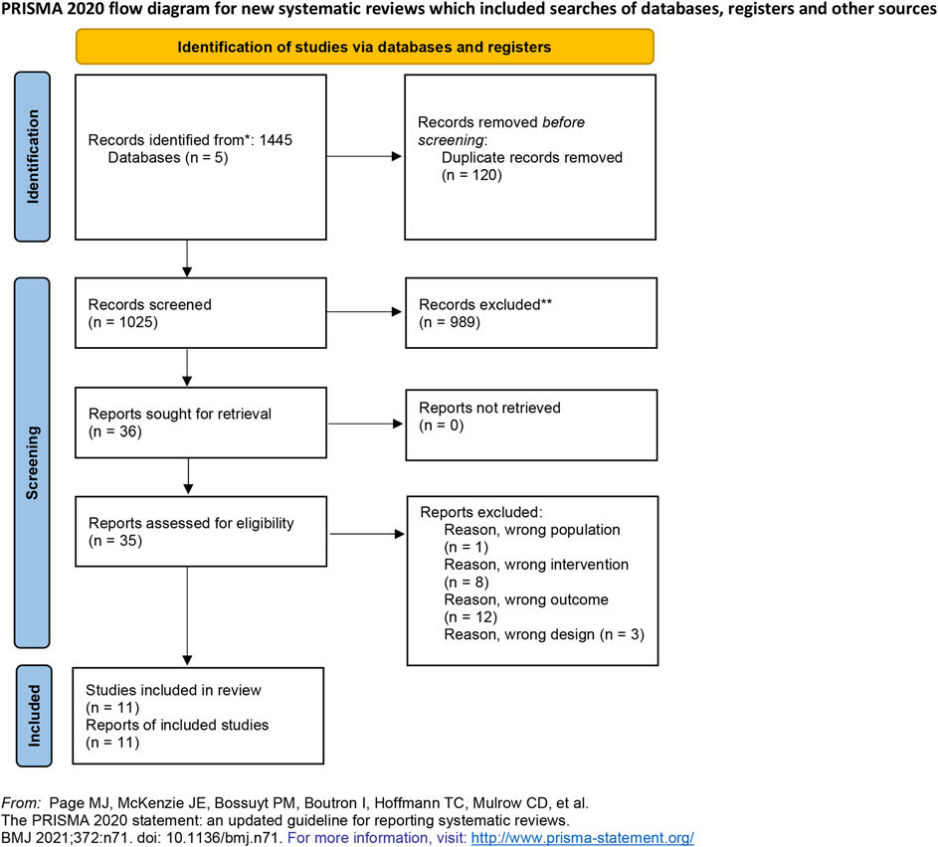
Prisma flow diagram.

From the literature search, 1445 documents could be identified. After removing 120 duplicates, the remaining papers were evaluated on the basis of title, abstract, and full text as described in the flowchart presented in Figure 1. No full text was added by Google Scholar search. A total of 11 articles were included for qualitative and quantitative analysis.^17–27^ The excluded full-text list was presented as Supplementary Datas S1 and S2.

### ROB analysis for included studies

**FIG. 2.**
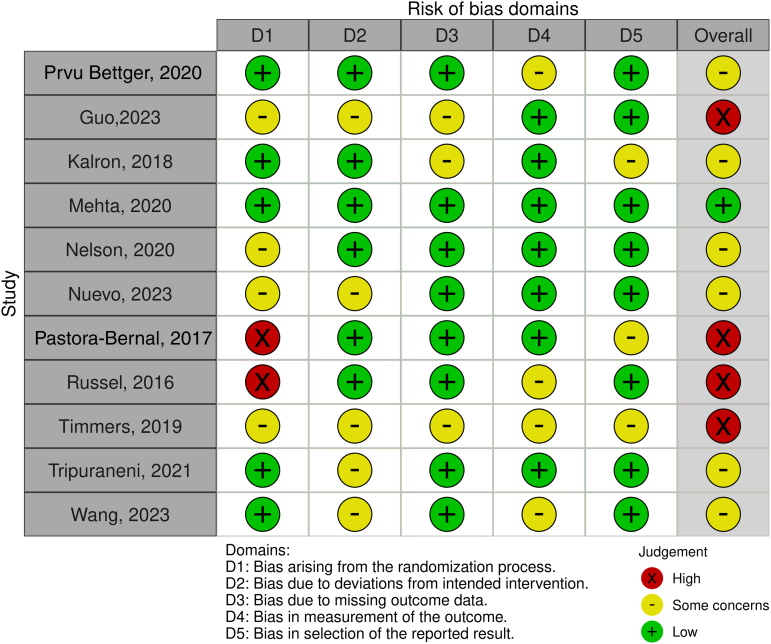
ROB assessment. ROB, risk of bias.

In the overall ROB analysis, only one article^20^ was given a low risk, six were assigned moderate risk^17,19,21,22,26,27^ and four were tagged as high risk.^18,23–25^ With respect to the assessment of deviations from the intended treatment, management of missing data and the choice of outcomes, ROB was low for most articles. In the domain regarding the randomization process, four articles^18,21,22,25^ showed an intermediate ROB while, a high ROB was found in two articles.^23,24^ The quality assessment of the articles is summarized in Figure 2.

### Qualitative data summary

The basic characteristics of the articles are presented in Table 2. The size of the sample in the articles varied widely, ranging from a minimum of 18 to a maximum of 337. The use of telerehabilitation was, for the most part, implemented for joint replacement surgery patients, particularly after knee replacement.^17,19,20,23–25^ The other surgical procedures considered are hip replacement,^18–20,22^ subacromial decompression,^22^ and anterior cruciate ligament.^18^

**Table 2.** Characteristics of the Included Studies

Author, year	Simple size (I/C)	Surgery, age (mean)	Intervention treatment	Control treatment	Outcome
Prvu Bettger et al. (2020)^17^	306 (153/153)	TKA, 65.3	Virtual Exercise Rehabilitation Assistant. Asynchrony modality. The telehealth therapist provided remote clinician oversight (one visit per week)	Usual care group followed their care team's recommendations. Not supervised	Gait speed (10 m gait speed) Pain (NRS 0–10) Knee function (KOOS)
Guo et al. (2023)^18^	125 (62/63)	ACL surgery, 28.9	Rehabilitation Cloud Platform. Asynchrony exercises.	Paper version of the rehabilitation plan, not supervised.	Pain (VAS) Functional scale (IKDC)
Kalron et al. (2018)^19^	32 (15/17)	Surgical hip operation, 66.5	Video platform for therapy software program. Asynchrony modality	Exercise booklet, not supervised	Functional test (TUG)
Mehta (2020)^20^	242 (118/124)	TKA, THA 66 (median)	Remote activity monitoring with automatic feedback. Asynchrony exercises.	Usual care (unclear)	Functional test (TUG)
Nelson et al. (2020)^21^	70 (35/35)	THA, 64.5	Identical intervention program, but delivered by telerehabilitation Asynchrony exercises.	Paper-based home exercise program, not supervised	Functional test (TUG) Functional scale (HOOS)
Nuevo et al. (2023)^22^	45 (23/22)	TKA, 68.2	Telerehabilitation system, ReHub^®^ was composed of two main parts: a web platform and an inertial motion sensor. Asynchrony exercises.	Leaflet detailing a program of five exercises, not supervised.	Functional test (TUG) Pain (VAS) Functional scale (WOMAC)
Pastora-Bernal et al. (2018)^23^	18 (9/9)	ASD, 52.2 (median)	Telerehabilitation via web-based exercise programs and videoconferencing. Synchrony modality.	Usual face-to-face physical therapy	Pain Score (0–15) Functional scale (Constant Murley Test)
Russel et al. (2011)^24^	20 (10/10)	TKA, 67	One telerehabilitation session per week, 45 min. Synchrony modality.	One face-to-face rehabilitation session per week, 45 min	Functional test (TUG) Pain (VAS) Functional scale (WOMAC)
Timmers et al. (2019)^25^	213 (114/99)	TKA, 65.2	All patients had access to an app, patients could unlock day-to-day information by entering a personal code. Asynchrony modality	All patients had access to an app, patients only received weekly, not supervised	Pain (NRS) Functional scale (KOOS)
Tripuraneni et al. (2021)^26^	337 (153/184)	TKA, 65.1	Mymobility platform is a patient–physician web-based interface. Patients download a mobile application and receive daily reminders. Asynchrony modality	Standard of care, patient education, and postoperative physical therapy, not supervised	Functional scale (KOOS)
Wang et al. (2023)^27^	86 (43/43)	THA, TKA (68.0)	Rehabilitation program delivered via mobile application (WeChat). Asynchrony exercises.	Verbal explanation and a take-home pamphlet for a rehabilitation regime that patients can follow at home, not supervised.	Pain (NRS) Functional scale (HOOS/KOOS)

ACL, anterior cruciate ligament; ASD, arthroscopic sub-acromial decompression; I/C, intervention/control; IKDC, International Knee Documentation Committee; KOOS, Knee Injury and Osteoarthritis Outcome Score; NRS, numeric rating scale; THA, total hip arthroplasty; TKA, total knee arthroplasty; TUG, Time Up and Go; VAS, visual analogue scale; WOMAC, Western Ontario and McMaster Universities Arthritis Index.

The most frequent method used to deliver telerehabilitation involved apps and software that were used to monitor the activity performed independently by the patient with feedback from the physiotherapist who set and gradually modified the exercise regime. In some cases, physiotherapy treatment was administrated through videoconferencing meetings. The most frequent control treatment was the home exercise that the patient performed alone without supervision. Only two studies^23,24^ used face-to-face treatment as a comparator.

The outcomes were assessed using multiple rating scales designed to measure the patient's motor performance and their perception of functional recovery and pain. Individual studies did not always explore all of these aspects but were, in most cases, limited to one or two of these outcomes. Functional scales based on patient perception were the most frequent choice.

### Quantitative data summary

**FIG. 3.**
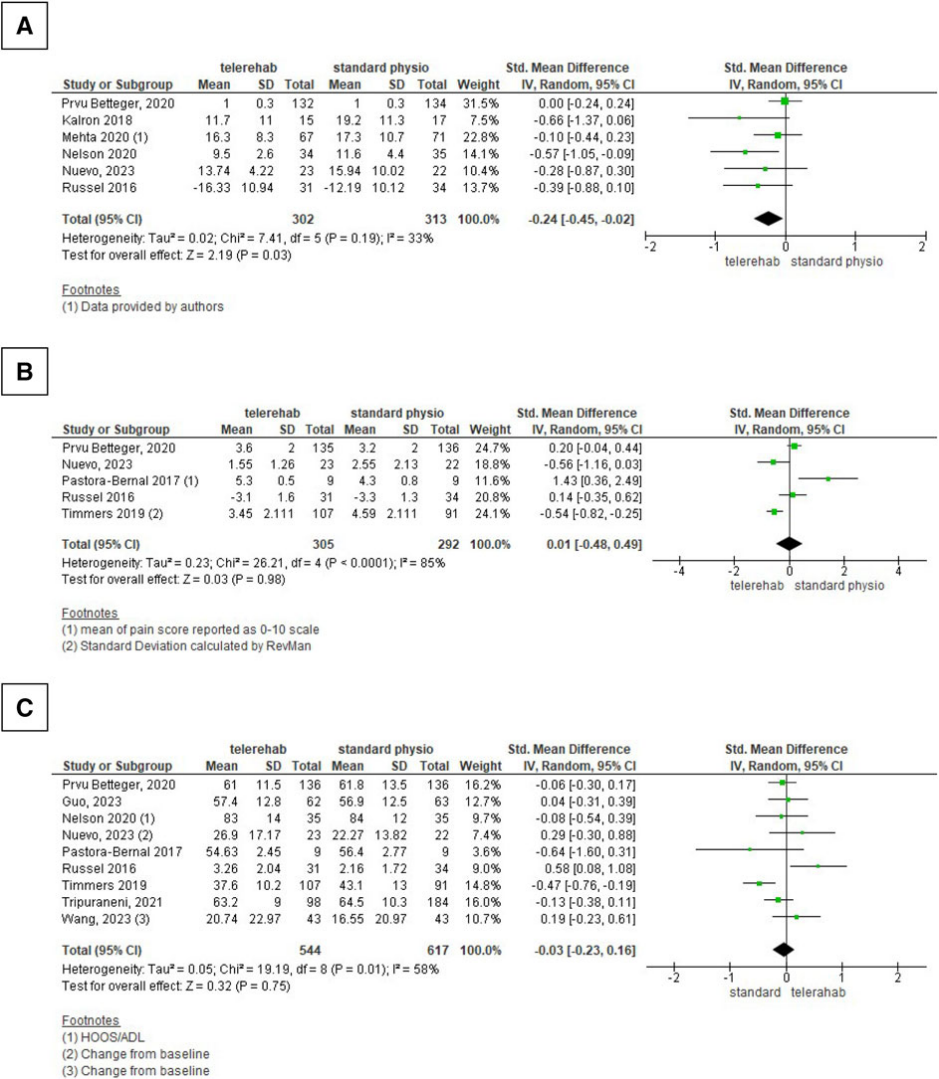
**(A)** Pooled estimation for motor performance; **(B)** for pain score; **(C)** for functional recovery.

The meta-analysis conducted (Fig. 3) regarding patient-achieved motor performance involved six studies for a total of 615 patients. The results obtained in the intervention group showed an improvement of −0.24 (from −0.45 to −0.02) compared to the control group (*p* = 0.03). Analysis of heterogeneity was moderate (I2 = 33%) (Fig. 3A).

Subgroups analysis related to the type of outcome used and type of surgery revealed a significant reduction in heterogeneity, down to 0% and 15%, respectively. Analyzing the five studies that used TUG as an outcome showed an average difference between the intervention and control groups of 2.21 sec (−3.57 to −0.86) in favor of the intervention group. The meta-analysis conducted comparing telerehabilitation with home-based treatment performed without supervision confirmed the same result in terms of MD, significance and heterogeneity. The sensitivity analysis with respect to the methodological quality didn't add explanation about the level of heterogeneity of the studies included.

**FIG. 4.**
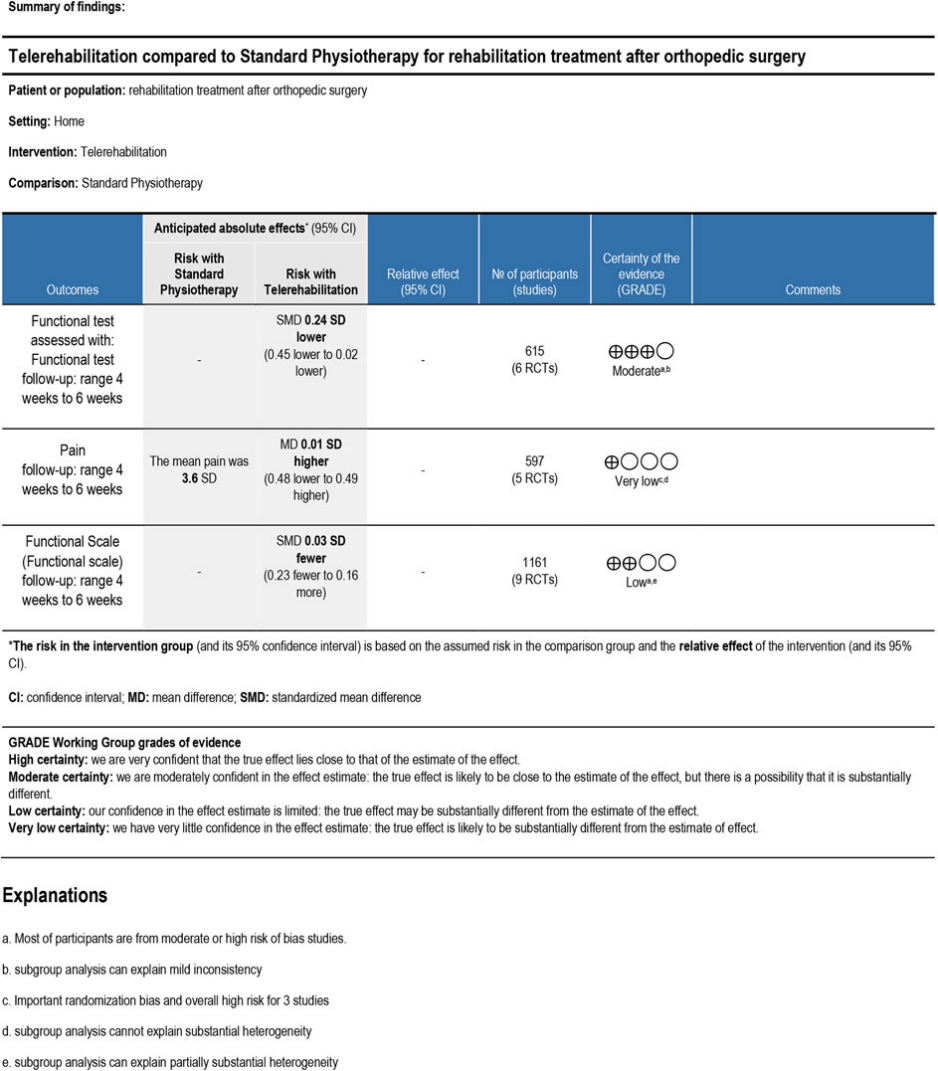
Summary of findings.

All subgroups and sensitivity analyses conducted were presented as Supplementary Datas S1 and S2. The certainty of the evidence assessed according to the GRADE method was moderate (Fig. 4).

Pain analysis included five studies and revealed a difference of 0.01 points (−0.48 to 0.49) in favor of the standard physiotherapy group with a statistical significance of 0.98 and substantial heterogeneity (I2 = 85%) (Fig. 3B). The subanalysis conducted on those studies using knee replacement as type of surgery and home-based physiotherapy as comparator showed a difference in favor of intervention group but same level of heterogeneity. The sensitivity analysis with respect to the methodological quality included only two studies, and it was not conducted. The certainty of evidence was very low (Fig. 4).

Based on patient-oriented rating scales, evaluation of functional recovery included nine studies that evaluated a total 1,161 patients and showed an effect estimate favoring standard physiotherapy of 0.03 (−0.023 to 0.16), statistical nonsignificance, (*p* = 0.75) and moderate heterogeneity (58%) (Fig. 3C). Subgroups analysis related to the type of outcome used, type of surgery, and home-based physiotherapy as comparator showed the same results. The sensitivity analysis with respect to the methodological quality included five studies and showed an effect estimate favoring intervention of 0.11 points, statistical nonsignificance (*p* = 0.12), and low heterogeneity (0%) The certainty of evidence was low (Fig. 4).

It was not possible to perform the publication bias analysis as the number of 10 articles included in a single meta-analysis was not reached.

## Discussion

Systematic review of the literature and the corresponding meta-analysis show that for orthopedic surgery patients, there were differences between the rehabilitation treatment administered via a digital system and standard physiotherapy in terms of motor performance at the first check-up 4–6 weeks after surgery. In the TUG evaluation, there was a statistically significant difference in favor of the group: the estimated improvement was 2.22 sec with a confidence interval (CI) ranging from 3.63 to 0.80. This result is confirmed when were analyzed studies reporting home-based physiotherapy as a comparative treatment. These values were close to the Minimal Detectable Changes reported in the literature for TUG in a population of patients who had undergone TKA and THA, where it was 2.27 and 1.67, respectively.^28,29^ As regards pain and functional recovery, the estimate of the effect favored the control group, while in both cases, the differences were not statistically and clinically significant.

The table summarizing the results (Fig. 4) showed results that ranged from a moderate level of certainty for functional performance outcomes, to a very low level for pain and to low level for patient-perceived functional recovery. The low quality of the studies taken into account resulted in downgrading of the evidence for all outcomes considered. The inconsistency in the results led to downgrading of the results for pain and functional recovery, thus highlighting the need for good-quality studies with more standardized outcomes. Larger studies with smaller CIs are needed to better define whether significant differences in the proposed interventions actually exist.

In presenting a review in the orthopedic postoperative setting, Pastora-Bernal et al.^10^ ended by confirming that an exhaustive conclusion could not be drawn regarding the effectiveness of telerehabilitation. However, they did emphasize that, in patients undergoing hip and knee replacements, the effectiveness of telerehabilitation was more pronounced. The authors did not conduct a meta-analysis of the selected articles but limited themselves to a qualitative summary of the data.

In line with available literature,^30,31^ in the current systematic review, few studies compared telerehabilitation and face-to-face treatment, and it was not possible to conducted a specific meta-analysis about it. Jiang et al.^30^ encourages telerehabilitation for patients after TKA because of its comparable pain control and better improvement of functional recovery as compared to face-to-face rehabilitation but meta-analysis conducted included only two studies. Conclusive evidence that telerehabilitation is comparable to standard face-to-face treatment would have required planning proper equivalence studies which, in turn, require much larger study samples. For some studies, the numbers were chosen by convention, or calculated according to the principle of noninferiority of the outcome starting from the minimum clinical difference. Furthermore, the type of intervention and control should be better standardized.

The COVID-19 pandemic gave strong impetus to the use of telerehabilitation as a tool to address the health situation which had, in many settings and for prolonged periods of time, made it impossible to provide face-to-face rehabilitation treatment.^3^ This has been reflected in an increase in scientific production in this area, but, to date, no overriding conclusions have been possible.

At the current state of the art, the studies conducted are mainly aimed at patients undergoing joint, hip and, in most cases, knee replacement surgery. Few data are available on treatment pathways for patients undergoing other types of orthopedic surgery.

### Limits

The results produced by this meta-analysis must be analyzed in the light of certain considerations. First, we must consider the variability in the method of telerehabilitation intervention: different timing, different approaches, and different evaluation outcomes. The use of different outcomes seems to result in increasingly heterogeneous meta-analyses. In the cases for which analysis was proposed, using the same outcome voided such heterogeneity. When planning further studies in this area, this aspect must be taken into account.

Second, it was not possible to perform an adequate analysis of the Publication Bias, because there were not at least 10 articles available for the construction of the funnel plot and the main publication platforms of the research protocols were not investigated.

## Conclusion

For joint replacement patients, treatment conducted via telerehabilitation appears able to provide levels of recovery better to that achieved through home-based physiotherapy in terms of motor performance. In contrast, conclusive evidence that telerehabilitation is comparable to standard face-to-face treatment are not available. Further equivalence studies with different types of orthopedic patients are needed to support broader use of telerehabilitation.

## Supplementary Material

Supplemental data

Supplemental data

## Abbreviations Used

ACL: anterior cruciate ligament
ASD: arthroscopic sub-acromial decompression
CI: confidence interval
HOOS: Hip Knee Disability and Osteoarthritis Outcome Score
I/C: intervention/control
IKCD: International Knee Documentation Committee
KOOS: Knee injury and Osteoarthritis Outcome Score
MD: mean difference
MeSH: Medical Subject Heading
NRS: numeric rating scale
PEDro: Physiotherapy Evidence Database
RADE: Grading of Recommendations Assessment, Development and Evaluation
ROB: risk of bias
THA: total hip arthroplasty
TKA: total knee arthroplasty
TUG: Time Up and Go
VAS: visual-analogue scale
WOMAC: Western Ontario and McMaster Universities Osteoarthritis Index

## References

[1] National Library of medicine—PUBMED, MeSH database web [Internet]. Available from: https://www.ncbi.nlm.nih.gov/mesh/?term=telerehabilitation [Last accessed: June 2022].

[2] Cox NS, Dal Corso S, Hansen H, et al. Telerehabilitation for chronic respiratory disease. Cochrane Database Syst Rev 2021;29;1(1):CD013040. 10.1002/14651858.CD013040.pub2 PMC8095032 33511633

[3] Subedi N, Rawstorn JC, Gao L, et al. Implementation of telerehabilitation interventions for the self-management of cardiovascular disease: Systematic review. JMIR Mhealth Uhealth 2020;27;8(11):e17957. 10.2196/17957 PMC7732711 33245286

[4] Tchero H, Tabue Teguo M, Lannuzel A, et al. Telerehabilitation for stroke survivors: Systematic review and meta-analysis. J Med Internet Res 2018;20(10):e10867. 30368437 10.2196/10867 PMC6250558

[5] Suso-Martí L, La Touche R, Herranz-Gómez A, et al. Effectiveness of telerehabilitation in physical therapist practice: An umbrella and mapping review with meta-meta-analysis. Phys Ther 2021;101(5):pzab075. 33611598 10.1093/ptj/pzab075 PMC7928612

[6] Seron P, Oliveros MJ, Gutierrez-Arias R, et al. Effectiveness of telerehabilitation in physical therapy: A rapid overview. Phys Ther 2021;101(6):pzab053. 33561280 10.1093/ptj/pzab053 PMC7928601

[7] Agostini M, Moja L, Banzi R, et al. Telerehabilitation and recovery of motor function: A systematic review and meta-analysis. J Telemed Telecare 2015;21(4):202–213. 25712109 10.1177/1357633X15572201

[8] Mani S, Sharma S, Omar B, et al. Validity and reliability of Internet-based physiotherapy assessment for musculoskeletal disorders: A systematic review. J Telemed Telecare 2017;23(3):379–391. 27036879 10.1177/1357633X16642369

[9] Turolla A, Rossettini G, Viceconti A, et al. Musculoskeletal physical therapy during the COVID-19 pandemic: Is telerehabilitation the answer? Phys Ther 2020;100(8):1260–1264. 32386218 10.1093/ptj/pzaa093 PMC7239136

[10] Pastora-Bernal JM, Martín-Valero R, Barón-López FJ, et al. Evidence of benefit of telerehabitation after orthopedic surgery: A systematic review. J Med Internet Res 2017;19(4):e142. 28455277 10.2196/jmir.6836 PMC5429438

[11] Liberati A, Altman DG, Tetzlaff J, et al. The PRISMA statement for reporting systematic reviews and meta-analyses of studies that evaluate healthcare interventions: Explanation and elaboration. BMJ 2009;339:b2700. 19622552 10.1136/bmj.b2700 PMC2714672

[12] Ouzzani M, Hammady H, Fedorowicz Z, et al. Rayyan—A web and mobile app for systematic reviews. Syst Rev 2016;5:210; doi: 10.1186/s13643-016-0384-4 27919275 PMC5139140

[13] Sterne JAC, Savovic J, Page MJ, et al. RoB 2: A revised tool for assessing risk of bias in randomised trials. BMJ 2019;366:l4898. 31462531 10.1136/bmj.l4898

[14] McGuinness LA, Higgins JPT. Risk-of-bias VISualization (robvis): An R package and Shiny web app for visualizing risk-of-bias assessments. Res Synth Methods 2021;12:55–61. 32336025 10.1002/jrsm.1411

[15] Sterne JA, Sutton AJ, Ioannidis JP, et al. Recommendations for examining and interpreting funnel plot asymmetry in meta-analyses of randomised controlled trials. BMJ 2011;343:d4002. 21784880 10.1136/bmj.d4002

[16] McMaster University. GRADEpro guideline development tool [software]. Developed by Evidence Prime, Inc., 2020. Available from: https://gradepro.org [Last accessed: November 2023].

[17] Prvu Bettger J, Green CL, Holmes DN, et al. Effects of virtual exercise rehabilitation in-home therapy compared with traditional care after total knee arthroplasty: VERITAS, a randomized controlled trial. J Bone Joint Surg Am 2020;102(2):101–109. 31743238 10.2106/JBJS.19.00695

[18] Guo Y, Li D, Wu YB, et al. Mobile health-based home rehabilitation education improving early outcomes after anterior cruciate ligament reconstruction: A randomized controlled clinical trial. Front Public Health 2023;10:1042167. 36711410 10.3389/fpubh.2022.1042167 PMC9877440

[19] Kalron A, Tawil H, Peleg-Shani S, et al. Effect of telerehabilitation on mobility in people after hip surgery: A pilot feasibility study. Int J Rehabil Res 2018;41(3):244–250. 29794545 10.1097/MRR.0000000000000296

[20] Mehta SJ, Hume E, Troxel AB, et al. Effect of remote monitoring on discharge to home, return to activity, and rehospitalization after hip and knee arthroplasty: A randomized clinical trial. JAMA Netw Open 2020;3(12):e2028328. 33346847 10.1001/jamanetworkopen.2020.28328 PMC7753899

[21] Nelson M, Bourke M, Crossley K, et al. Telerehabilitation is non-inferior to usual care following total hip replacement—A randomized controlled non-inferiority trial. Physiotherapy 2020;107:19–27. 32026820 10.1016/j.physio.2019.06.006

[22] Nuevo M, Rodríguez-Rodríguez D, Jauregui R, et al. Telerehabilitation following fast-track total knee arthroplasty is effective and safe: A randomized controlled trial with the ReHub^®^ platform. Disabil Rehabil 2023;5:1–11. 10.1080/09638288.2023.2228689 37403684

[23] Pastora-Bernal JM, Martín-Valero R, Barón-López FJ, et al. Telerehabilitation after arthroscopic subacromial decompression is effective and not inferior to standard practice: Preliminary results. J Telemed Telecare 2018;24(6):428–433. 28449618 10.1177/1357633X17706583

[24] Russell TG, Buttrum P, Wootton R, et al. Internet-based outpatient telerehabilitation for patients following total knee arthroplasty: A randomized controlled trial. J Bone Joint Surg Am 2011;93(2):113–120. 21248209 10.2106/JBJS.I.01375

[25] Timmers T, Janssen L, van der Weegen W, et al. The effect of an app for day-to-day postoperative care education on patients with total knee replacement: Randomized controlled trial. JMIR Mhealth Uhealth 2019;7(10):e15323. 31638594 10.2196/15323 PMC6914303

[26] Tripuraneni KR, Foran JRH, Munson NR, et al. A smartwatch paired with a mobile application provides postoperative self-directed rehabilitation without compromising total knee arthroplasty outcomes: A randomized controlled trial. J Arthroplasty 2021;36(12):3888–3893. 34462184 10.1016/j.arth.2021.08.007

[27] Wang Q, Hunter S, Lee RL, et al. The effectiveness of a mobile application-based programme for rehabilitation after total hip or knee arthroplasty: A randomised controlled trial. Int J Nurs Stud 2023;140:104455. 36821950 10.1016/j.ijnurstu.2023.104455

[28] Yuksel E, Kalkan S, Cekmece S, et al. Assessing minimal detectable changes and test-retest reliability of the timed up and go test and the 2-minute walk test in patients with total knee arthroplasty. J Arthroplasty 2017;32(2):426–430.. 27639305 10.1016/j.arth.2016.07.031

[29] Yuksel E, Unver B, Kalkan S, et al. Reliability and minimal detectable change of the 2-minute walk test and Timed Up and Go test in patients with total hip arthroplasty. Hip Int 2021;31(1):43–49. 31928090 10.1177/1120700019888614

[30] Jiang S, Xiang J, Gao X, et al. The comparison of telerehabilitation and face-to-face rehabilitation after total knee arthroplasty: A systematic review and meta-analysis. J Telemed Telecare 2018;24(4):257–262. 28027679 10.1177/1357633X16686748

[31] Shukla H, Nair SR, Thakker D. Role of telerehabilitation in patients following total knee arthroplasty: Evidence from a systematic literature review and meta-analysis. J Telemed Telecare 2017;23(2):339–346. 26843466 10.1177/1357633X16628996

