# Supplementary material for: The Effectiveness of Telerehabilitation for Functional Recovery After Orthopedic Surgery: A Systematic Review and Meta-Analysis
Source: Telemed Rep. 2024 Mar 27;5(1):78–88. doi: 10.1089/tmr.2023.0057 (PMC10979691; doi:10.1089/tmr.2023.0057)
Supplement: Supplemental data [file Suppl_DataS1.docx]

List of excluded studies:

| **Study** | **Reason** |
| --- | --- |
| Aldemir K, Gürkan A. The effect of pedometer-supported walking and telemonitoring after disc hernia surgery on pain and disability levels and quality of life. Int J Nurs Pract. 2021 Apr;27(2):e12917. doi: 10.1111/ijn.12917. Epub 2021 Feb 16. PMID: 33594720. | Wrong interventiom |
| Ardern CL, Kvist J; BANG Trial Group. BAck iN the Game (BANG) - a smartphone application to help athletes return to sport following anterior cruciate ligament reconstruction: protocol for a multi-centre, randomised controlled trial. BMC Musculoskelet Disord. 2020 Aug 8;21(1):523. doi: 10.1186/s12891-020-03508-7. PMID: 32770983; PMCID: PMC7414541. | Wrong design |
| Bini SA, Mahajan J. Clinical outcomes of remote asynchronous telerehabilitation are equivalent to traditional therapy following total knee arthroplasty: A randomized control study. J Telemed Telecare. 2017 Feb;23(2):239-247. doi: 10.1177/1357633X16634518. Epub 2016 Jul 9. PMID: 26940798. | Wrong outcome (follow-up timing) |
| Correia FD, Nogueira A, Magalhães I, Guimarães J, Moreira M, Barradas I, Teixeira L, Tulha J, Seabra R, Lains J, Bento V. Home-based Rehabilitation With A Novel Digital Biofeedback System versus Conventional In-person Rehabilitation after Total Knee Replacement: a feasibility study. Sci Rep. 2018 Jul 26;8(1):11299. doi: 10.1038/s41598-018-29668-0. PMID: 30050087; PMCID: PMC6062628. | Wrong outcome (follow-up timing) |
| Eichler S, Salzwedel A, Rabe S, Mueller S, Mayer F, Wochatz M, Hadzic M, John M, Wegscheider K, Völler H. The Effectiveness of Telerehabilitation as a Supplement to Rehabilitation in Patients After Total Knee or Hip Replacement: Randomized Controlled Trial. JMIR Rehabil Assist Technol. 2019 Nov 7;6(2):e14236. doi: 10.2196/14236. PMID: 31697239; PMCID: PMC6873150. | Wrong outcome (follow-up timing) |
| Gianola S, Stucovitz E, Castellini G, Mascali M, Vanni F, Tramacere I, Banfi G, Tornese D. Effects of early virtual reality-based rehabilitation in patients with total knee arthroplasty: A randomized controlled trial. Medicine (Baltimore). 2020 Feb;99(7):e19136. doi: 10.1097/MD.0000000000019136. PMID: 32049833; PMCID: PMC7035049. | Wrong outcome (follow-up timing) |
| Harmelink KEM, Zeegers AVCM, Tönis TM, Hullegie W, Nijhuis-van der Sanden MWG, Staal JB. The effectiveness of the use of a digital activity coaching system in addition to a two-week home-based exercise program in patients after total knee arthroplasty: study protocol for a randomized controlled trial. BMC Musculoskelet Disord. 2017 Jul 5;18(1):290. doi: 10.1186/s12891-017-1647-5. PMID: 28679400; PMCID: PMC5498982. | Wrong disign |
| Hou J, Yang R, Yang Y, Tang Y, Deng H, Chen Z, Wu Y, Shen H. The Effectiveness and Safety of Utilizing Mobile Phone-Based Programs for Rehabilitation After Lumbar Spinal Surgery: Multicenter, Prospective Randomized Controlled Trial. JMIR Mhealth Uhealth. 2019 Feb 20;7(2):e10201. doi: 10.2196/10201. PMID: 30785406; PMCID: PMC6404639. | Wrong outcome (follow-up timing) |
| Koo KI, Park DK, Youm YS, Cho SD, Hwang CH. Enhanced Reality Showing Long-Lasting Analgesia after Total Knee Arthroplasty: Prospective, Randomized Clinical Trial. Sci Rep. 2018 Feb 5;8(1):2343. doi: 10.1038/s41598-018-20260-0. PMID: 29402908; PMCID: PMC5799299. | Wrong intervention |
| Kline PW, Melanson EL, Sullivan WJ, Blatchford PJ, Miller MJ, Stevens-Lapsley JE, Christiansen CL. Improving Physical Activity Through Adjunct Telerehabilitation Following Total Knee Arthroplasty: Randomized Controlled Trial Protocol. Phys Ther. 2019 Jan 1;99(1):37-45. doi: 10.1093/ptj/pzy119. PMID: 30329126; PMCID: PMC6314331. | Wrong design |
| Lee ASY, Shu-Hang Yung P, Ong MT, Lonsdale C, Wong TWL, Siu PM, Hagger MS, Chan DKC. Effectiveness of a theory-driven mHealth intervention in promoting post-surgery rehabilitation adherence in patients who had anterior cruciate ligament reconstruction: A randomized clinical trial. Soc Sci Med. 2023 Oct;335:116219. doi: 10.1016/j.socscimed.2023.116219. Epub 2023 Sep 9. PMID: 37716185. | Wrong outcome (follow-up timing) |
| Levinger P, Hallam K, Fraser D, Pile R, Ardern C, Moreira B, Talbot S. A novel web-support intervention to promote recovery following Anterior Cruciate Ligament reconstruction: A pilot randomised controlled trial. Phys Ther Sport. 2017 Sep;27:29-37. doi: 10.1016/j.ptsp.2017.06.001. Epub 2017 Jun 8. PMID: 28822956. | Wrong intervention |
| Martinez-Rico S, Lizaur-Utrilla A, Sebastia-Forcada E, Vizcaya-Moreno MF, de Juan-Herrero J. The Impact of a Phone Assistance Nursing Program on Adherence to Home Exercises and Final Outcomes in Patients Who Underwent Shoulder Instability Surgery: A Randomized Controlled Study. Orthop Nurs. 2018 Nov/Dec;37(6):372-378. doi: 10.1097/NOR.0000000000000501. PMID: 30451774. | Wrong intervention |
| Moffet H, Tousignant M, Nadeau S, Mérette C, Boissy P, Corriveau H, Marquis F, Cabana F, Belzile ÉL, Ranger P, Dimentberg R. Patient Satisfaction with In-Home Telerehabilitation After Total Knee Arthroplasty: Results from a Randomized Controlled Trial. Telemed J E Health. 2017 Feb;23(2):80-87. doi: 10.1089/tmj.2016.0060. Epub 2016 Aug 16. PMID: 27529575. | Wrong outcome (follow-up timing) |
| Moffet H, Tousignant M, Nadeau S, Mérette C, Boissy P, Corriveau H, Marquis F, Cabana F, Ranger P, Belzile ÉL, Dimentberg R. In-Home Telerehabilitation Compared with Face-to-Face Rehabilitation After Total Knee Arthroplasty: A Noninferiority Randomized Controlled Trial. J Bone Joint Surg Am 2015; 15;97(14):1129-41. | Wrong outcome (follow-up timing) |
| Palm KB, Blazar PE, Manna JC, Serig AS, Phillips EA, Bay CP, Casey EJ, Earp BE. Feasibility, effectiveness and patient satisfaction of telerehabilitation after thumb carpometacarpal arthroplasty and reverse total shoulder arthroplasty: A pilot study. J Telemed Telecare. 2023 Aug;29(7):521-529. doi: 10.1177/1357633X21999578. Epub 2021 Mar 5. PMID: 33673751. | Wrong outcome  (follow-up timing) |
| Piqueras M, Marco E, Coll M, Escalada F, Ballester A, Cinca C, Belmonte R, Muniesa JM. Effectiveness of an interactive virtual telerehabilitation system in patients after total knee arthoplasty: a randomized controlled trial. J Rehabil Med. 2013 Apr;45(4):392-6. doi: 10.2340/16501977-1119. PMID: 23474735. | Wrong outcome (follow-up timing) |
| Rothgangel A, Braun S, Winkens B, Beurskens A, Smeets R. Traditional and augmented reality mirror therapy for patients with chronic phantom limb pain (PACT study): results of a three-group, multicentre single-blind randomized controlled trial. Clin Rehabil. 2018 Dec;32(12):1591-1608. doi: 10.1177/0269215518785948. Epub 2018 Jul 16. PMID: 30012007. | Wrong intervention |
| Shim GY, Kim EH, Lee SJ, Chang CB, Lee YS, Lee JI, Hwang JH, Lim JY. Postoperative rehabilitation using a digital healthcare system in patients with total knee arthroplasty: a randomized controlled trial. Arch Orthop Trauma Surg. 2023 Oct;143(10):6361-6370. doi: 10.1007/s00402-023-04894-y. Epub 2023 May 2. PMID: 37129691. | Wrong outcome (follow-up timing) |
| Tao G, Miller WC, Eng JJ, Lindstrom H, Imam B, Payne M. Self-directed usage of an in-home exergame after a supervised telerehabilitation training program for older adults with lower-limb amputation. Prosthet Orthot Int. 2020 Apr;44(2):52-59. doi: 10.1177/0309364620906272. Epub 2020 Mar 1. PMID: 32114933. | Wrong population |
| Tousignant M, Moffet H, Boissy P, Corriveau H, Cabana F, Marquis F. A randomized controlled trial of home telerehabilitation for post-knee arthroplasty. J Telemed Telecare. 2011;17(4):195-8. doi: 10.1258/jtt.2010.100602. Epub 2011 Mar 11. PMID: 21398389. | Wrong outcome (follow-up timing) |
| van Dijk-Huisman HC, Weemaes ATR, Boymans TAEJ, Lenssen AF, de Bie RA. Smartphone App with an Accelerometer Enhances Patients' Physical Activity Following Elective Orthopedic Surgery: A Pilot Study. Sensors (Basel). 2020 Aug 2;20(15):4317. doi: 10.3390/s20154317. PMID: 32748876; PMCID: PMC7436024. | Wrong intervention |
| Vesterby MS, Pedersen PU, Laursen M, Mikkelsen S, Larsen J, Søballe K, Jørgensen LB. Telemedicine support shortens length of stay after fast-track hip replacement. Acta Orthop. 2017 Feb;88(1):41-47. doi: 10.1080/17453674.2016.1256939. Epub 2016 Nov 16. PMID: 28097941; PMCID: PMC5251263. | Wrong intervention |
| Wang J, Tong Y, Jiang Y, Zhu H, Gao H, Wei R, Que X, Gao L. The effectiveness of extended care based on Internet and home care platform for orthopaedics after hip replacement surgery in China. J Clin Nurs. 2018 Nov;27(21-22):4077-4088. doi: 10.1111/jocn.14545. Epub 2018 Aug 1. PMID: 29851157. | Wrong outcome (follow-up timing) |
