# Supplementary material for: The Effectiveness of Telerehabilitation for Functional Recovery After Orthopedic Surgery: A Systematic Review and Meta-Analysis
Source: Telemed Rep. 2024 Mar 27;5(1):78–88. doi: 10.1089/tmr.2023.0057 (PMC10979691; doi:10.1089/tmr.2023.0057)

**Supplementary material**

A1 Subgroup analysis for TUG outcome


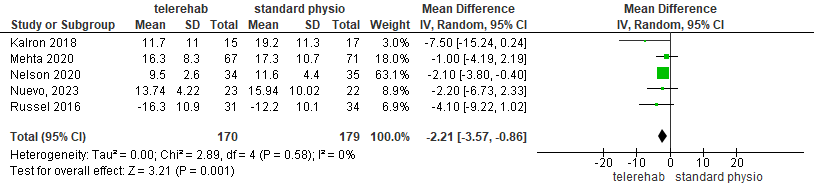


A2 Sensitivity analysis related to methodological quality for studies that assessed motor performance


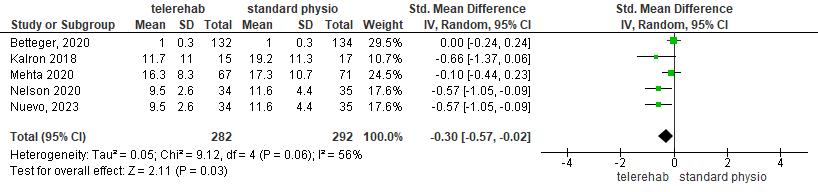


A3 Subgroup analysis for knee replacement and motor performance


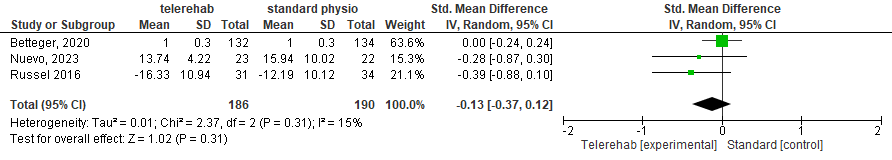


A4: Subgroup analysis for TUG outcome and home-based physiotherapy ad comparator


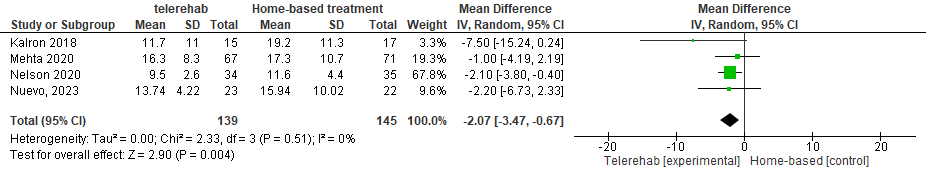


B3 Subgroup analysis for knee replacement and pain score


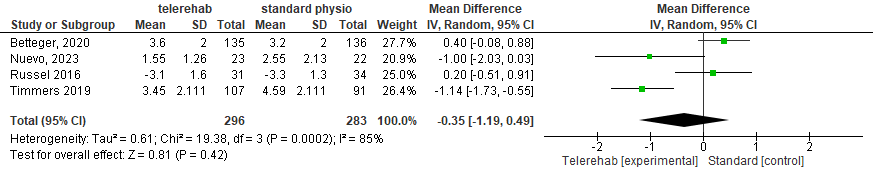


B4: Subgroup analysis for pain score and home-based physiotherapy as comparator


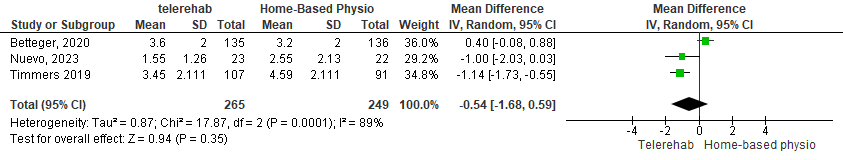


C1 Subgroup analysis for HOOS/KOOS outcome


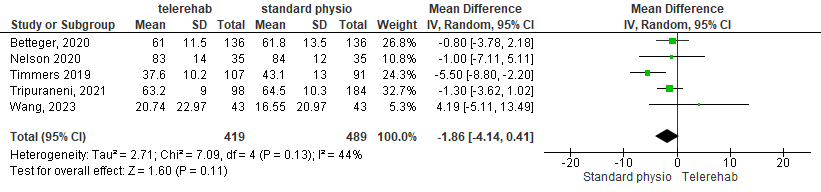


C2 Sensitivity analysis related to methodological quality for studies that assessed the functional recovery


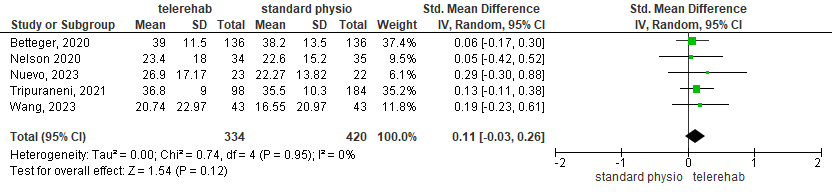


C3 Subgroup analysis for knee replacement and functional recovery


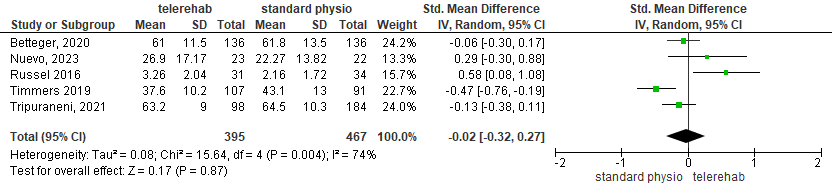


C4 Subgroup analysis for functional recovery and home-based physiotherapy as comparator


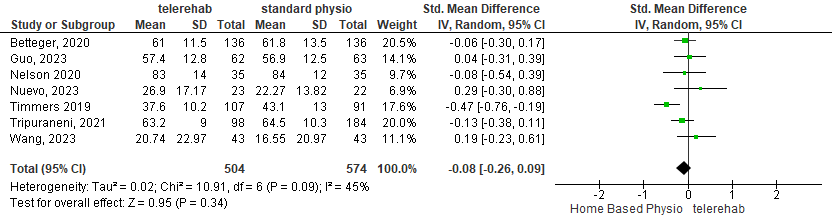

Supplement: Supplemental data [file Suppl_DataS2.docx]
